# Credit risk contagion of supply chain finance: An empirical analysis of supply chain listed companies

**DOI:** 10.1371/journal.pone.0306724

**Published:** 2024-08-27

**Authors:** Xinpeng Geng, Bing Han, Debao Yang, Junren Zhao

**Affiliations:** 1 Shipping Economics and Management College, Dalian Maritime University, Dalian, China; 2 Collaborative Innovation Center for Transport Studies, Dalian Maritime University, Dalian, China; University of Almeria: Universidad de Almeria, SPAIN

## Abstract

With the gradual rise of the supply chain financial model and the expansion of scale, credit risk and contagion effects are gradually strengthened as business and financial links between upstream and downstream enterprises in the supply chain. The traditional credit risk contagion assessment model based on the financial status of an enterprise and the pledging of fixed assets has been unable to meet the basic needs of modern supply chain companies and financial institutions for risk control. Therefore, this paper introduces the Cox-Copula model to comprehensively assess a company’s financial situation and the business health of upstream and downstream companies in the supply chain from the perspective of actual transactions between companies and fixed asset pledges. The study found that credit risk has a contagion effect in supply chain enterprises, and this contagion effect of credit risk has certain dynamic characteristics. At the same time, it was found that the impacts of macroeconomic factors and microfinance factors on credit risk contagion of supply chain finance have differences in the two dimensions of degree and direction of action.

## 1. Introduction

Credit risk contagion is described as a phenomenon of the debtor’s default strength increasing due to the breach of another debtor, regardless of the relationship between the enterprises [1]. Compared with credit risk contagion in traditional financing practices with individual firms, the contagion is assessed in terms of the overall supply chain but still focused on corporate financial data in the recent studies. For instance, the effectiveness of estimating credit rating transition matrices was studied by using sequence-based clustering on historical credit rating sequences [2]. A financial risk prevention model for supply chain enterprises was proposed based on financial data from listed companies of China to reduce supply chain risks and minimize the impact of corporate risks on the supply chain [3]. A Grey correlation model was applied to assess the credit risk of supply chain finance in the Chinese home appliance industry with some performance indicators that represent profitability, solvency, operational capability, and development capability [4]. The online supply chain financial credit risk assessment system was established based on a hybrid model chain and took upstream enterprises in the supply chain of construction industry in China as an example [5]. In fact, all of these recent results did not consider the actual transactions occurring within the supply chain at all but for the financial data provided by these enterprises. In China, with the development of the supply chain finance model, more and more small and medium-sized enterprises(SMEs) have solved the problem wherein it is difficult to get a loan due to the introduction of the real transactions into credit risk assessment. According to the McKinsey report, from 2010 to 2014, the global supply chain financial business has achieved almost 20% growth and is expected to maintain a growth rate of 15% in 2019. However, with the rapid advancement of the supply chain financial business, the issue of credit risk has become increasingly prominent. Taking account receivables financing as an example, the credit sales ratio of SMEs in China in 2016 was still as high as 78%. The average account period increased from 57 days in 2014 to 68 days, an increase of 19%. Sixty-eight percent of the companies surveyed encountered customer defaults, 26.3% of which had an average default period of over 90 days, and 15.9% of enterprises had an average default period of over 150 days. Credit risk has become a major obstacle to the advancement of the supply chain financial business, as well as a major challenge for the industry and academia to realize a new model of industrial-financial integration.

The causes of credit risk are related to the relatively late development of China’s credit economy, the underdevelopment of commercial credit, and a weak awareness of credit among the people [6]. As a result, accounts receivables that could be recovered through legal channels have become bad debts and bad debt losses. At the same time, with the credit risk problem, the breadth of infectivity further increased the complexity and difficulty of risk control. Often, one company’s default causes other associated companies to default or even go bankrupt, forming a domino-style credit risk contagion effect [7,8]. Therefore, this study’s objective is to examine the credit risk measurement and contagion effects between upstream and downstream companies in the supply chain by constructing a Cox-Copula model and conduct an empirical analysis of the credit risk contagion of the supply chain enterprises from different industries. Correspondingly, there are three research questions raised. Firstly, what is the mechanism behind the contagion effect of supply chain credit risk, and how to characterize the contagious effect of credit risk? Secondly, what factors contribute to the contagion effect of supply chain credit risk? Thirdly, what are the considerations for selecting the appropriate model to assess the credit risk and contagion effects of upstream and downstream companies in the supply chain?

This paper studies the risk measurement issues involved in supply chain finance using the Cox model and the contagious effects of credit risk using the Copula model. To verify the validity of the model and provide theoretical and practical guidance for supply chain financial companies and related financial institutions further improving risk control, we use data from Oriental Eastcom Peace and Eastern Communications. The supply chain of the Internet software industry is also used in this paper. Upstream and downstream listed companies Tianxia Wisdom and Haoyun Technology, as research objects, study and analyze the influence of industry factors on the contagion effect of credit risk. The Cox model, a semi-parametric model in a multi-factor survival analysis method proposed by Cox in 1972, is used to analyze the influence of many factors on the survival time of the study subjects. For a new credit risk assessment model that uses actual transactions as collateral, the Cox model has strong continuity. Without establishing a model for each survival time, the time variable can be analyzed as a continuous one, which can help improve prediction performance caused by oversampling. The Copula model is used to characterize credit risk from the perspective of the interdependence between member companies in the supply chain. The actual transactions between companies upstream and downstream of the supply chain are used as research objects to provide a new way to study corporate credit risk and contagion perspective. The default forecast model with contagion effect in this paper measures the interdependence of defaults between companies more accurately and provides a reference for identifying credit risk contagion issues among enterprises, and financial institutions’ credit risk management.

## 2. Literature review

At present, the research on the credit risk of supply chain finance has aroused widespread attention and achieved many results regarding a variety of aspects such as risk control, assessment methods, and member collaboration [9]. As this study examines the contagion effect of credit risk in supply chain finance. Thus, in this section, several related topics are reviewed including the factors and mechanisms, the measurement methods and the contagion effect of credit risk in supply chain finance.

### 2.1 Factors and mechanisms influencing credit risk

In the research on influencing factors and mechanisms of supply chain financial credit risk, different factors and mechanisms have been discussed in previous studies, such as financial characteristics, collaborative factors, trust model and so on [10–19]. For example, Le studied the relationship between core enterprises and SMEs under the supply chain finance model, i.e., information sharing to reach consensus, collaborative management of various aspects, and joint business in cross-border trade through the influence of cultural factors [2]. If companies had a large inventory and high interest rate restrictions, introducing supply chain finance will have a beneficial effect. On the contrary, it might cause a company to fall into a financial crisis. Caniato studied the issue of the introduction of supply chain finance to the sustainable development and efficiency improvement of members in an entire industrial chain and found that the discount rate of payables and period factors had significant influence [10]. In addition, retailer credit, the working financial process, transaction credit, electronic trading platforms, open account credit, bank loans, and account lead-time also had a significant impact on members of the entire industry chain. Huff studied the factors that affect the healthy operation of supply chain finance between a single monopoly manufacturer and an oligopolistic retailer with competitive factors and proposed that transaction credit had a positive effect on its healthy operation and a negative effect on the accounting period [11]. Şenay investigated the multiregional supply chain credit risk measured by abnormal credit default swaps (CDS) spreads and US–China supply chain networks under the impact of COVID-19 [12]. With the development of new technologies such as big data, cloud computing, and blockchain, Jiang proposed a trust transitivity model based on blockchain to evaluate the trust of small and medium-sized manufacturing enterprises (SMMEs) so as to give full play to the value-added and transmission effect of the manufacturing industry chain relieve the financing difficulties for SMMEs [13]. Ma identified the most important elements in supply chain collaboration from the perspective of financial service providers by employing interpretive structural modelling to model the relationship between collaborative factors and understand the importance of each factor [14]. Xiao utilized entropy weighting to construct independent variables and applied logistic regression to explore the impact of financing enterprises, core enterprises, asset positions during financing, blockchain platforms, and supply chain operations on credit risk [15]. Martins observed that lending to firms connected through the supply chain conveys valuable information to banks by using unique administrative data on firm-to-firm and bank-to-firm lending [16]. Beka explored key factors influencing Supply Chain Finance adoption including information sharing, collaboration, digitization, and financial institution involvement and studies the impact of these factors on improving the supply chain effectiveness [17]. Wu employed company-level data to develop a machine learning framework with gradient boosting decision trees and investigated the impact of supply chain information on predicting corporate credit ratings [18].

### 2.2 Credit risk measurement

The research on credit risk measurement methods of supply chain finance can be traced back to the expectation and variance measurement method proposed by American economist Markowitz in 1952, thus creating a quantitative era of risk measurement. Shatanawi further introduced the Conditional Value-at-Risk (CVaR) high-dimensional combination model with norm constraints that greatly diversifies tail risk when measuring credit risk [19]. Arif studied the dynamics of an infectious disease in a population has a stochastic nature [20]. Because its objective function is a quadratic loss function, it features the characteristics of continuous, smooth, etc., characteristics and has good ductility, which can disperse the tail risk, making its evaluation result more robust. Mou constructed a supply chain financial credit risk evaluation system that included 4 risk categories and 10 risk factors, then the risk identification model constructed by means of a fuzzy analytic hierarchy process was used to analyze core enterprise credit risks in supply chain finance [21]. Xia built a credit risk evaluation system based on four common-used machine learning algorithms to measure the corporate credit risk by using small and medium-sized enterprises in the Chinese stock market from 2015 to 2020 as research samples [22]. Fayyaz developed a reinforced prediction model that used a social network analysis to enrich prediction attributes and supervised machine learning approaches to support assessing credit risks of actors in a supply chain finance network [23]. Wang developed new forecast models based on machine learning techniques and applied these new models to predict credit risk of SMEs in China based on financial information, operation information, innovation information, and negative events as predictors [24]. Belhadi introduced an innovative hybrid ensemble machine learning method for predicting credit risk in the context of SMEs’ investments in Agriculture 4.0 within supply chain finance based on the Rotation Forest and Logit Boosting algorithms [25]. Zhang used the firefly algorithm support vector machine (FA-SVM) to assess credit risk in supply chain finance with the higher classification accuracy and lower error rates compared to traditional SVM methods [26]. Wang constructed the commercial bank online supply chain financial credit risk assessment index system and conducted an empirical analysis using the nonlinear least-squares support-vector machines(LS-SVM) model compared with the logistic regression model in classification accuracy [27]. Li proposed a robust credit risk model for Supply Chain Finance using PCA-GA-SVM which involves dimensionality reduction by principal component analysis (PCA) and SVM parameter optimization by the genetic algorithm (GA) [28]. The results demonstrated superior generalization ability over traditional SVM and GA-SVM methods.

### 2.3 Credit risk contagion effect

Regarding the research on supply chain financial credit risk contagion model, Mizgier proposed a default infection model based on the factor model, which divided the companies with the same credit rating into contagion supply chain companies and infected supply chain companies and proved the existence of credit risk contagion effects through stress tests [29]. Xie proposed a new credit risk assessment method based on graph theory and fuzzy preference theory with two supply chain credit risk types: "individual credit risk" and "credit risk contagion" to reveal the transmission effects of related credit risks within the supply chain [30]. Xie also investigated the dual-channel financing model where the retailer and the manufacturer can seek loans from bank, and the retailer can also obtain trade credit from the manufacturer [8,31,32]. Under dual-channel financing, it showed that the contagion effect of credit risk is higher when the retailer prioritizes repaying the bank loan rather than repaying trade credit, and the contagion effect also depends on the bank loan ratio and production costs. Chen explored the transmission speed and steady state of credit risk when the supply chain finance network is affected by the impact of epidemic to have a more complete understanding of the ability of supply chain finance to resist risks [33]. Mu constructed a bank-firm credit matching network model utilizing an Agent-Based Model (ABM) framework and reinforcement learning algorithm to investigate interaction behaviors and the mechanism of credit risk network contagion by considering the heterogeneity of behavioral rules, learning rules, and interaction rules [34]. Ma proposed a SEIR-based model to analyze the equilibrium point and stability of this model, as well as find the threshold value for risk contagion and simulate the model dynamically to analyze the influence of the parameters [35]. Spatareanu analyzed the direct and indirect impact of banks’ default risk on firms’ default risk in the U.K., further examined the impact the trade credit and contract specificity on the propagation of default risk [36]. It revealed that increases in banks’ default risk from the banking crisis of 2007–2008 propagated strongly to U.K. non-financial firms via supply chains.

In addition, because the Copula function has flexibility and universality in describing dependence between variables, it has a wide range of applications in the study of the contagion effect of credit risk. Darwish used a layered Archimedes-Copula function to examine the contagious effect of credit risk based on many customer behavior (profile) parameters such geographical locations, usage frequency, and book balance [37,38]. Tian proposed a generalized autoregressive conditional heteroskedasticity (GARCH) copula quantile regression model to capture the downside and upside tail dependence between oil price change and stock market returns at different risk levels and estimated the downward and upward risk spillovers from oil to stock markets [39]. Li adopted the copula-conditional value at risk (Copula-CoVaR) model to examine the risk spillover effects within the supply chain system in China during the COVID-19 crisis [40]. Abodayeh introduced dynamic macro-factors to maximize the effectiveness of the model in detecting fraudulent transactions regardless the presence of any data imbalance [41]. Abakha examined the novel time-varying Markov-switching copula model, re-evaluating the empirical relationship between risk and returns in 15 international stock markets from December 1994 to August 2020 [42]. Carta used a Markov intensity Copula function and found that how the proposed model outperforms several state-of-the-art solutions, both in terms of ensemble models and classification approaches [43]. Wu utilized a time-varying copula-CoVaR approach to investigate the transmission of risks within the Chinese financial system [44].

The study provides a basis for the supply chain financial risk measurement and contagious problem research, including the factors affecting credit risk, evaluation models, and contagion models. This paper will be different from traditional risk assessment models based on fixed asset pledges or based on actual transactions. As a research object, the supply chain financial business of collateral is introduced. A semi-parametric Cox model that can analyze survival time continuously and has no special restrictions on covariates is introduced. The Copula model that captures the correlation between variables is simultaneously used to construct the Cox-Copula model and study the credit risk and contagion effects among members of the supply chain.

## 3. Research design

### 3.1 Process description

**Fig 1:**
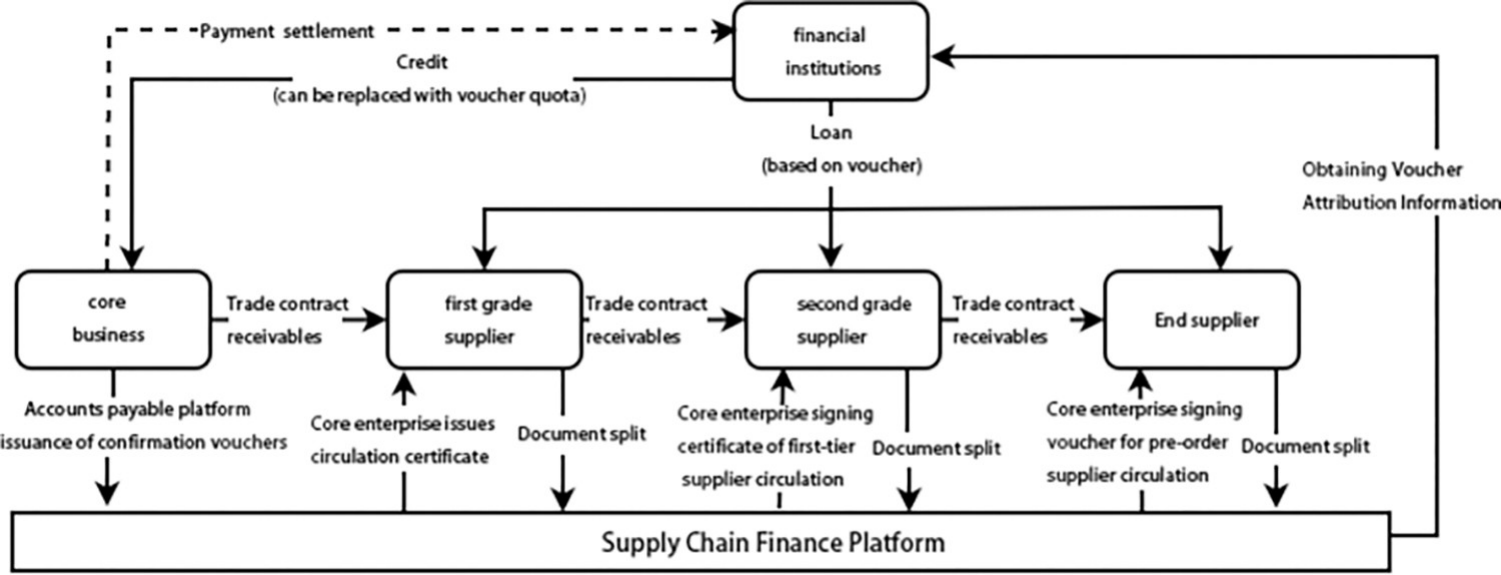
Schematic diagram of supply chain financial services.

The essence of supply chain finance is a financial service provider. For the assets with poor liquidity owned by an enterprise during the operation of various channels in the supply chain, the future cash flow generated by the assets is used as a direct repayment source and closed capital operations are also used. The model provides personalized financial service solutions and its specific operational process is illustrated in Fig 1. Financial institutions such as banks provide credit to core companies and provide loans to primary, secondary, and terminal suppliers to enhance the overall synergy of the supply chain. In the process of issuing, splitting, and circulating accounts receivables and vouchers among members at all levels of the supply chain, due to the frequent occurrence of credit defaults and risk contagion events, relevant models need to be introduced to measure these credit risk and contagion effects. Different from the pledge financing of traditional financial institutions, supply chain financial business financing takes actual transactions as collateral. In the actual operational process, banks and other financial institutions use supply chain financial platforms to verify transactions with core companies and other upstream and downstream companies. The enterprise’s authenticity and reliability, possible future cash flows, and financial status are used to assess any corresponding credit default risk issues.

An important application of credit risk research is to construct default forecast models. The research on default forecast models can be traced back to the univariate analysis model [45]. The second-generation default forecasting model is based mainly on a quantitative response such as typical logit models and other non-parametric methods, such as cluster analysis and neural networks. The current research is based on duration analysis, the Cox risk proportion model introduced in this paper. This paper first uses the Cox risk ratio model to measure the credit risk of supply chain companies, draws a trend chart with time as the independent variable and default risk as the dependent variable, and then evaluates the contagion effect of its credit risk by introducing the Copula model. Therefore, this paper proposes the following hypotheses and further verifies them through the empirical analysis described in the next section.

Hypothesis 1. Between upstream and downstream companies exists the dependency relationship with synchronized credit risk trends, which indicates potential risk transmission in the supply chain.

Hypothesis 2. The credit risk contagion effect among different supply chain industries exhibits different dynamic characteristics.

### Assumptions

The study of credit risk contagion of supply chain finance is based on the following basic assumptions:

1. Recognizing the limitations in information and cognitive processing, all financial institutions, as well as upstream and downstream companies make decisions based on the best available information and within their capacity.
2. All the suppliers and core companies will repay the loans as much as possible without engaging in fraudulent activities.
3. All financial institutions, as well as upstream and downstream companies, are risk-averse, leading them to finance suppliers with stable credit histories and extend credit terms cautiously to maintain a lower risk profile.

### 3.3 Set-up model

The Cox model can measure the impact of indicators or indicator systems on the company’s credit risk contagion. The basic form is:

$$h(t|X)=h_{0}(t)\exp(X^{T}\beta )$$ (1)

Where *h*(*t*|*X*) represents the default probability of a supply chain company with covariate X at time t, that is, there is no default risk before time t, but the instantaneous risk of default at time t. *β* = (*β*_1_,…,*β*_*p*_)^*T*^ is the risk regression coefficient, which is also the variable that is most concerned in this paper. $e^{\beta_{j}}$ represents the relative risk of the company’s default, that is, if *β*_*j*_>0, the corresponding covariate *X*_*j*_ increases by 1 unit, and the relative risk of the company’s default increases by $e^{\beta_{j}}$ times. Conversely, the relative risk of a company defaulting is reduced by $1—e^{\beta_{j}}$. *h*_0_(*t*) is the benchmark risk rate, which indicates the probability that a company will default when all the covariates affecting the company’s credit risk are zero. Since *h*(*t*|*X*) cannot be observed, it only represents instantaneous risk and cannot be effectively measured, so the following survival function is established to represent the probability that the company has no default at time t:

$$S(t|X)=\exp[-\int_{0}^{t}h(x)dx]=S_{0}{\left(t\right)}^{\exp({X_{i}}^{T}\beta )}$$ (2)

Where $S_{0}(t)=\exp(-\int_{0}^{t}h_{0}(u)du)$ is the benchmark function. The solution of *β* is usually obtained by maximizing the partial likelihood function *L*(*β*). Set *n* companies with *K* observations with different survival times, *n-k* final inspection values, and K observations with survival times. *t*_1_<…<*t*_*k*_, the defaulted companies correspond to the *i =* 1,…, *k* individuals. If the set of defaults given at time *t*_*i*_ is *R*_*i*_,*R*_*i*_ represents the set of companies that are still observing at time *t*_*i*_ (including companies that defaulted at time *t*_*i*_), then under the condition that a company default occurs at time *t*_*i*_, The probability that it happens to be the ith company is:

$$\frac{h_{0}(t_{i})\exp(\beta^{T}X)}{\sum_{l\in R_{i}}h_{0}(t_{i})\exp(\beta^{T}X_{l})}=\frac{\exp(\beta^{T}X)}{\sum_{l\in R_{i}}h_{0}(t_{i})\exp(\beta^{T}X_{l})}$$ (3)

Suppose there are k defaults in n companies, use *t*_(1)_<…<*t*_(*k*)_ to indicate the sequence time of the company defaults (assuming that there is no "knot"), *X*_(*i*)_ to the explanatory value of the explanatory variable of company *i*, and *R*(*t*_(*i*)_) to the company’s normal financial time is at least *F* Set of all companies, then the partial likelihood function is:

$$L(\beta )=\prod_{i=1}^{k}\frac{e^{{X^{T}}_{\left(i\right)}\beta }}{\sum_{j\in R(t_{\left(i\right)})}e^{{X^{T}}_{\left(j\right)}\beta }}$$ (4)

The partial likelihood function expressed in Eq (4) is different from the general likelihood function, but the estimate $\overset{\wedge }{\beta }$ (which is called the maximum partial likelihood estimation of β) that obtains the maximum value β of Eq (4) generally has a likelihood function the asymptotic nature of can be treated the same as maximum likelihood estimation. Let *T*_*i*_ be the survival time of the i-th company, *C*_*i*_ be the censoring time Zimin (*T*_*i*_,*C*_*i*_), and the censoring index *δ*_*i*_ = *I*(*T*_*i*_≤*C*_*i*_). According to Breslow’s idea, considering *H*_0_(∙)’s "least information" non-parametric model, *H*_0_(*t*) has a jump hj at the observation default time $t_{j}^{0}$, namely: $H_{0}(t)=\sum_{i=1}^{N}h_{j}I({t_{j}}^{0}\leq t)$, $H_{0}(X_{i})=\sum_{i=1}^{N}h_{j}I(i\in ℜ({t_{j}}^{0}))$. The strength of a company’s default *λ*_*t*_ = Λ(*X*_*t*_) can be expressed as a function of the covariate *H*, denoted *J*, and the strength of the company’s exit due to non-default reasons is *α*_*t*_ = A(*X*_*t*_) This considers a credit risk default strength model and withdrawal strength model, namely $\lambda (x_{t};\mu_{0};\mu )=e^{\mu_{0}+\mu^{T}x_{t}}$ and $\alpha (x_{t};v_{0};v)=e^{v_{0}{+v}^{T}x_{t}}$. Under the double random assumption of default strength and withdrawal strength, the company’s conditional probability of default in the next s years is:

$$p(T<t+s|F_{t})=q(X_{t},s)=E(\int_{t}^{t+s}e^{-\int_{t}^{z}(\lambda (u)+\alpha (u))du}\lambda (z)dz|X_{t})$$ (5)

Among them, represents the time before the company defaults, and *F*_*t*_ represents the observation information up to time *t*. Given the covariate process *X*_*t*_, the time of default and the time of withdrawal are independent of each other. However, the double randomness assumption has great limitations when considering credit contagion. For example, the default and withdrawal of a large company occupying a certain market (or industry) may lead to the Default or withdrawal, that is, conditions are not independent between companies. Therefore, when micro-industry credit contagion is considered, a new default intensity model is introduced:

$$\lambda (t)=\exp(\delta +\alpha^{T}X_{\left(t\right)}+\beta^{T}Y_{\left(t\right)}+\gamma^{T}Z_{\left(t\right)})$$ (6)

However, credit risk contagion involves not only the correlation between linear variables, but also the edge and joint probability distribution of nonlinear variables. Thus, this paper mainly uses the kernel density estimation in the non-parametric method to determine the edge distribution of the Copula model.

The kernel density estimation method is a smooth differentiable estimation of the Copula function based on the functional form of the Kernel structure, and it avoids the prior assumption of the related structure of the edge distribution.The kernel density function Kij (x) is bounded and symmetric in R numbers and satisfies ∫Kij (x) dx = 1, then its original function is:

$$K_{i}(x,h)=\prod_{j=1}^{n}k_{tj}(\frac{x_{j}}{h_{j}}),t=1,2,\ldots ,m$$ (7)

Among them, h is a window width, and a diagonal matrix with hj as an element. h_j_ is a positive function of *T*, and when *T* tends to infinity, | h | + 1 / T | h | → 0. At this time, the estimated formulas for the edge probability density and edge cumulative distribution function of *Y*_*tj*_ at point *y*_*i*_ are:

$$\overset{\Lambda }{f_{j}}(y_{j})=\frac{1}{Th_{j}}\sum_{t=1}^{T}\sum_{j=1}^{n}k_{j}(\frac{y_{j}-y_{jt}}{h_{j}})$$ (8)

$$\overset{\Lambda }{F_{j}}(y_{tj})=\int_{-\infty }^{y_{tj}}\overset{\Lambda }{f_{j}}(x)dx$$ (9)

The estimated expressions of joint probability density function and joint cumulative distribution function of *Y*_*t*_ at point *y* = (*y*_1_,*y*_2_,…,*y*_*n*_) are:

$$\overset{\Lambda }{f_{j}}(y_{j})=\frac{1}{T|h|}\sum_{t=1}^{T}\sum_{j=1}^{n}k_{j}(\frac{y_{j}-y_{jt}}{h_{j}})$$ (10)

$$\overset{\Lambda }{F}(y)=\int_{-\infty }^{y_{1}}\int_{-\infty }^{y_{2}}\cdot \cdot \cdot \int_{-\infty }^{y_{n}}\overset{\Lambda }{f}(x)dx$$ (11)

The non-linear relationship portrayed by the Copula model is helpful for analyzing the contagion effects of upstream and downstream companies on extreme events at the tail, while minimizing the loss of contagionness to the entire supply chain operation. For the characterization of the tail dependence of the random variables X1 and X2, the Copula model is expressed by the upper-tail correlation coefficient *λ*_*U*_ and the lower-tail correlation coefficient *λ*_*L*_, and its expression is as follows:

$$\lambda_{U}=\underset{u\to 1^{-}}{\lim}\mathrm{Pr}[x_{1}>F^{-1}x_{1}(u)|x_{2}>F^{-1}x_{2}(u)]=\underset{u\to 1^{-}}{\lim}\frac{1-2u+C(u,u)}{1-u}$$ (12)

$$\lambda_{L}=\underset{u\to 0^{+}}{\lim}\mathrm{Pr}[x_{1}\leq F^{-1}x_{1}(u)|x_{2}\leq F^{-1}x_{2}(u)]=\underset{u\to 0^{+}}{\lim}\frac{C(u,u)}{u}$$ (13)

Among them, *λ*_*U*_,*λL*∈ [0,1], the upper-tail correlation coefficient *λ*_*U*_ (the lower-tail correlation coefficient *λ*_*L*_) describes that when the random variable X2 is greater than (less than or equal to) a certain critical value, the random variable X1 is also greater than (less than or equal to) the probability of the threshold. The time-varying Copula model can well describe the asymmetric tail correlation between random variables, so it has a wider application in the field of risk management. For example, Luo Changqing constructed a time-varying Copula model under jump-diffusion conditions to measure the tail dependence of credit risk between upstream and downstream enterprises in the supply chain. The specific definition is as follows:

$$C(u_{1},u_{2};\theta ,\delta )=\eta (\eta^{-1}(u_{1})+\eta^{-1}(u_{2}))=1-{\left[1-{\left(1-{\left(1-u_{1}\right)}^{\theta }\right)}^{-\delta }+{\left(1-{\left(1-u_{2}\right)}^{\theta }\right)}^{-\delta }-1^{-\frac{1}{\delta }}\right]}^{\frac{1}{\theta }}$$

among them,η(s) = 1-[1-(1+s)-1/δ]1/θ, θ≥1,δ>0, Upper tail correlation coefficient*λ*_*U*_ = 2−2^1/θ^, Bottom-tail correlation coefficient*λ*_*L*_ = 2^1/δ^. However, since the parameters θ, δ of the time-varying Copula model do not intuitively reflect the tail dependence relationship between random variables, and the tail dependence relationship is the starting point for studying the credit risk contagion problem between upstream and downstream companies in the supply chain, this article uses the tail. After the dependence coefficients *λ*_*U*_ and *λ*_*L*_ are transformed by the formulas θ = log2 / log (2-λ_U_) and δ = -log2 / logλ_L_, the function expression is as follows:

$$C(u_{1},u_{2};\lambda_{L},\lambda_{U})=1-{\left[1-{\left\{{\left[1-{\left(1-u_{1}\right)}^{\frac{\log2}{\log(2-\lambda_{U})}}\right)}^{\frac{\log2}{\log\lambda_{L}}}+{\left(1-{\left(1-u_{2}\right)}^{\frac{\log2}{\log(2-\lambda_{U})}}\right)}^{\frac{\log2}{\log\lambda_{L}}}-1\right\}}^{\frac{\log\lambda_{2}}{\log2}}\right]}^{\frac{\log(2-\tau_{U})}{\log2}}$$

The transformed time-varying Copula model can directly estimate the tail dependence coefficients λ_U_ and λ_L_. And by introducing covariates, it is possible to characterize the dynamic characteristics of tail-dependent relationships of random variables and their influencing factors. According to the covariant Copula method [46], this paper assumes that the marginal distributions of the random variables X1 and X2 are the same and follow the split-t distribution. By adjusting the four parameters of the split-t distribution, we can get the fitted results of the marginal distribution according to the data characteristics of the random variables X1 and X2. We introduce covariates to estimate the marginal distribution of the random variables X1 and X2. The basic idea is as follows:

$$\mu_{ij}=x{`}_{ij}\beta_{\mu_{j}}$$ (14)

$$\varphi_{ij}=\exp(x{`}_{ij}\beta_{\varphi_{j}})$$ (15)

$$\nu_{ij}=\exp(x{`}_{ij}\beta_{\nu_{j}})$$ (16)

$$\kappa_{ij}=\exp(x{`}_{ij}\beta_{\kappa_{j}})$$ (17)

Among them, μ, φ, ν, κ are position parameters, scale parameters, degrees of freedom parameters, and skewness parameters of the split-t distribution, respectively. *x*_*ij*_ is the ith observation of the covariate vector that estimates the marginal distribution of the *j*th random variable, and β is the covariate coefficient of the marginal distribution. Through the logit function, the covariate *x* is introduced into the upper-tail correlation coefficient *λ*_*U*_ and the lower-tail correlation coefficient *λ*_*L*_, thereby constructing a covariate dynamic time-varying Copula model. The relationship between the covariate and the tail-dependent coefficients *λ*_*U*_ and *λ*_*L*_ is as follows:

$$\lambda_{U}={l^{-1}}_{\lambda_{U}}(x`\beta_{\lambda_{U}})$$ (18)

$$\lambda_{L}={l^{-1}}_{\lambda_{L}}(x`\beta_{\lambda_{L}})$$ (19)

Among them, L (·) is a logit function, *x* is a covariate vector, and β is a parameter of the covariate x.

This paper uses the full Bayesian estimation method to estimate the model parameters. First, the selection rules for the covariate setting variables are as follows:

$$I_{j}={\{}_{0,if\;\beta =0}^{1,if\;\beta \neq 0}$$ (20)

Where *β*_*j*_ is the coefficient value of each j-th variable in the model. In this paper, we assume that the prior distributions of the constant term β_0_ and the slope term β are independent of each other and follow the normal prior distribution. According to the Bayesian principle, we decompose the prior joint distribution of the parameters into:

$$p(\beta_{0},\beta ,I)=p(\beta_{0})p(\beta |I)p(I)$$ (21)

Then, the Metropoolis-Hasting Gibbs sampling method is used to simultaneously update the parameters of the Copula function and the marginal distribution parameters. The Metropoolis-Hasting algorithm is used to estimate the covariate parameters and the variable selection probability (β, I). Finally, K-fold-out-of-sample log predictive score (LPS) is used as the criterion for predicting the accuracy of the model. LPS is defined as:

$$LPS=\frac{1}{K}\sum_{k=1}^{K}\log p(y_{d}|y_{-d},x)$$ (22)

Among them, *y*_*d*_ is a matrix of nd × p contains nd samples in the d-th test set, and y-d is a training set. If we assume that the samples are independent of each other given the parameters (β, I), according to the Bayesian principle:

$$p(y_{d}|y_{-d},x)=\int \prod_{i\in d}p(y_{i}|\{\beta ,I\},x_{i})p(\{\beta ,I\}|y_{-d})d\{\beta ,I\}$$ (23)

LPS can be obtained by calculating the average of log *p*(*y*_*d*_|*y*_−*d*_,*x*) in the test set.

## 4. Empirical analysis

### 4.1 Data description and indicator selection

Electronic information technology and Internet software are fiercely competitive industries and are more susceptible to the effects of credit risk contagion. Therefore, this article selects Eastcom Peace and Eastern Communications, listed companies in the supply chain of the electronic information technology industry, as the research objects to study their credit risks and contagion effects. Tianxia Wisdom and Haoyun Technology, listed companies in the Internet software industry’s supply chain, were selected as research objects to study and analyze the influence of industry factors on the contagion effects of credit risk. The sample period extends from the first quarter of 2008 to the fourth quarter of 2018, and each company has 44 time series samples. The financial data of the listed companies comes from the Guotai Junan (CSMAR) database and the macro-covariate data comes from the WIND database. Due to information disclosure and related laws and regulations, it is difficult to obtain relevant data for the emerging Internet software industry. Only 28 time series samples were collected for each company from the first quarter of 2012 to the fourth quarter of 2018. The innovation of research on the contagion of credit risk in supply chain finance is reflected in the selection of the indicators. It not only selects financial data that can measure the operational status of the research object, but also introduces the evaluation dimension of enterprise qualification to assess the synergetic effect of the supply chain, thereby ensuring that the evaluation results are more robust. Among them, data on corporate credit ratings and historical default rates of the company come from the National Enterprise Credit Information Publicity System (NECIPS), and the data of the manager’s credit records come from the personal credit information system (PCIPS). Pledged cashing capacity, cooperation frequency, and order completion number come from the international research company Isuppli Co. Ltd. Wang Xiaoyan (2018) proposed a composite Minimax Concave Penalty (cMCP) punishment group method for the credit default risk of supply chain companies and demonstrated the rationality and stability of credit default risk from the four dimensions of debt repayment ability, financial ratio structure, cash flow level, and qualification. Zhang Xinmin (2019) studied the selection of indicators under solvency and qualification and Yue Aidong (2019) discussed the selection of indicators for cash flow level and financial ratio structure. Therefore, the preliminary selection of indicators obtained through comprehensive screening and processing are shown in Tables 1 and 2.

**Table 1:** Macro-covariate description.

Variable name	Variable description	Mean	Standard deviation
X1 Consumer Price Index	Inflation rate proxy variable	2.675	2.146
X2 GDP growth rate	Economic Development Speed Agent Variable	8.872	1.635
X3 Broad money growth rate (M2 growth)	Money supply growth rate	16.845	4.125
X4 short term interest rate	Short-term loan interest rate within 6 months	5.519	0.522
X5 RMB/USD spot rate	RMB to USD exchange rate	6.856	0.721

**Table 2:** Description of the company’s micro-financial covariates.

First-level indicators	Second-level indicators			
Solvency	X6 Current ratio	X7 Interest coverage ratio	X8 Quick ratio	X9 Assets and liabilities
	X10 Equity coefficient	X11 Equity ratio	X12 Equity to debt ratio	
Financial ratio structure	X13 Current assets ratio	X14 Owner ratio	X15 Current debt ratio	X16 Operating debt ratio
	X17 Accounts receivable turnover	X18 Inventory turnover	X19 Accounts payable turnover rate	
Cash flow level	X20 Cash fit ratio	X21 Cash reinvestment ratio	X22 Cash meets investment rate	X23 Net cash flow per share
Stock investment value	X24 P / E ratio	X25 Market rate	X26 P / B ratio	X27 Q guest value
	X28 Book value	X29 Enterprise value multiple		

Solvency refers to the ability of an enterprise to repay long-term and short-term debt with its assets. The higher the solvency, the stronger the repayment ability of the supply chain company, and the lower its risk of default. Similarly, the cash flow level reflects the funding status of supply chain companies. The higher the cash flow, the more surplus funds the company has, and the lower its default risk. The financial ratio structure reflects the stability of a company’s daily operations. The healthier the structure, the lower the risk of corporate default. Enterprise qualification reflects the overall synergetic effect of supply chain enterprises, that is, the higher the qualification, the better the enterprise’s operating status and the lower the corresponding default risk.

Therefore, the research demonstrates that the financial ratio structure of the health of the daily operations of the company, the solvency and cash flow level of the capital situation, and enterprise qualification more comprehensively assess the credit default risk of supply chain companies.

### 4.2 COX model estimation results

This paper examines the model estimation results for Eastcom Peace and Eastern Communications in the electronic information technology industry to analyze credit risk and contagion effects, and further analyzes upstream and downstream supply chain companies in different industries, Tianxia Wisdom and Haoyun Technology to determine the extent of the contagion effect. This paper first selects the relevant data from Eastcom Peace and Eastern Communications using the cMCP-COX method to select 24 indicators, and finally selects six variables to enter into the model, involving four aspects, which are: X6, flow ratio under solvency; X11, equity ratio; X15, current debt ratio under the financial ratio structure; X22, cash meet investment rate under cash flow level; X25, cooperation frequency; and X29, order completion rate under enterprise qualification. The correlation matrix with variance inflation factor (VIF) of these variables are shown in Table 3. All the correlations are low and the variance inflation factors are well below the acceptable level of 10, implying no issue of multicollinearity.

**Table 3:** Correlation matrix and variance inflation factor.

	X6	X11	X15	X22	X25	X29
X6	1					
X11	-0.339	1				
X15	0.009	-0.254	1			
X22	-0.199	0.123	0.028	1		
X25	0.309	0.097	0.172	-0.029	1	
X29	0.217	0.243	-0.116	-0.102	0.263	1
VIF	5.438	3.679	3.84	1.243	5.08	7.283

The results of the factor analysis and parameter estimation based on the cMCP-COX method are shown in Table 4.

**Table 4:** COX regression results.

	β coefficient	Standard error	wald Square value	Degrees of freedom	P value	Relative risk
X1	-0.362	0.314	2.774	1	0.000215***	0.695
X2	-0.623	0.125	7.625	1	4.25e^-10^***	0.355
X3	-0.523	0.106	2.663	1	1.59e^-8^***	1.364
X4	0.621	0.098	3.125	1	0.001425**	0.356
X5	0.322	0.154	7.223	1	3.32e^-11^***	1.332
X6	0.378	0.102	2.862	1	0.000167***	1.459
X11	-0.874	0.156	8.534	1	6.42e^-11^***	0.367
X15	-0.322	0.103	3.024	1	0.001246**	0.712
X22	0.522	0.087	3.144	1	1.57e^-9^***	1.671
X25	-0.321	0.109	8.838	1	0.002298**	0.721
X29	-2.476	0.325	2.663	1	9.85e^-14^***	0.088

Note:

***, ***, * represent the values of α are 0.001, 0.01, 0.05 respectively, the same below.

After analyzing the results of the cMCP-COX regression model, the following conclusions can be drawn: current ratio and cash-satisfied investment ratios are dangerous factors; equity ratio, current debt ratio, market price ratio, and multiples of enterprise value are protective factors. The regression coefficient (β) of the current ratio is 0.378, which indicates that for each increase in the asset-liability ratio, the relative risk of the company’s default increases by 0.378 (1.459) times. The current ratio is the ratio of current assets to current liabilities. When the current ratio is high, the company’s solvency and profitability are poor, its stability is low, and its financial risk is high. The regression coefficient of the cash satisfaction investment rate is positive (0.522). For each additional unit of the cash satisfaction investment rate, the relative risk of a listed company defaulting will increase by 1.671 times. The regression coefficient of the current debt ratio is -0.321. For each additional unit, the relative risk of a listed company defaulting is reduced by 0.721 times. Similarly, the regression coefficients of ownership ratio, cooperation frequency, and order completion rate are all negative (-0.874, -0.322, and -2.476, respectively), indicating that these are protective factors, reducing the risk of default. For each additional unit, the company’s relative risk of default is reduced by 1-e^β^.

**Fig 2:**
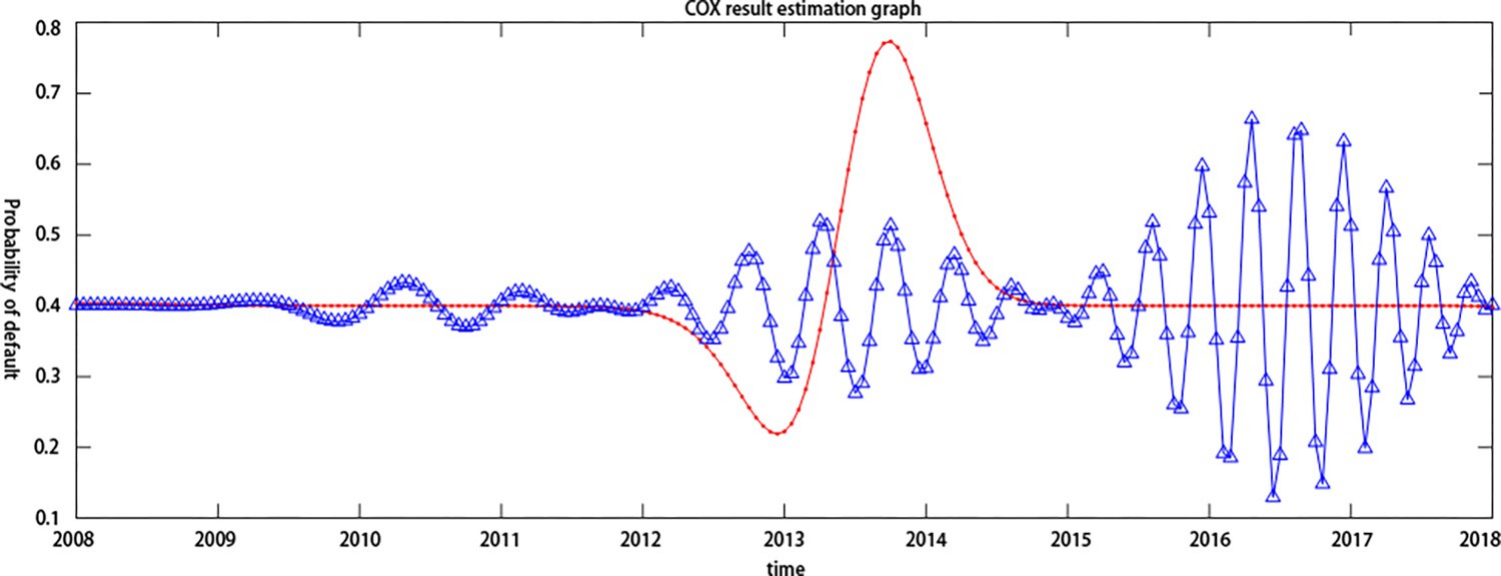
Comparison of credit risk synchronism in the electronic information technology industry. Note:Red Line: Eastcom Peace;Blue line: Eastern Communications.

**Fig 3:**
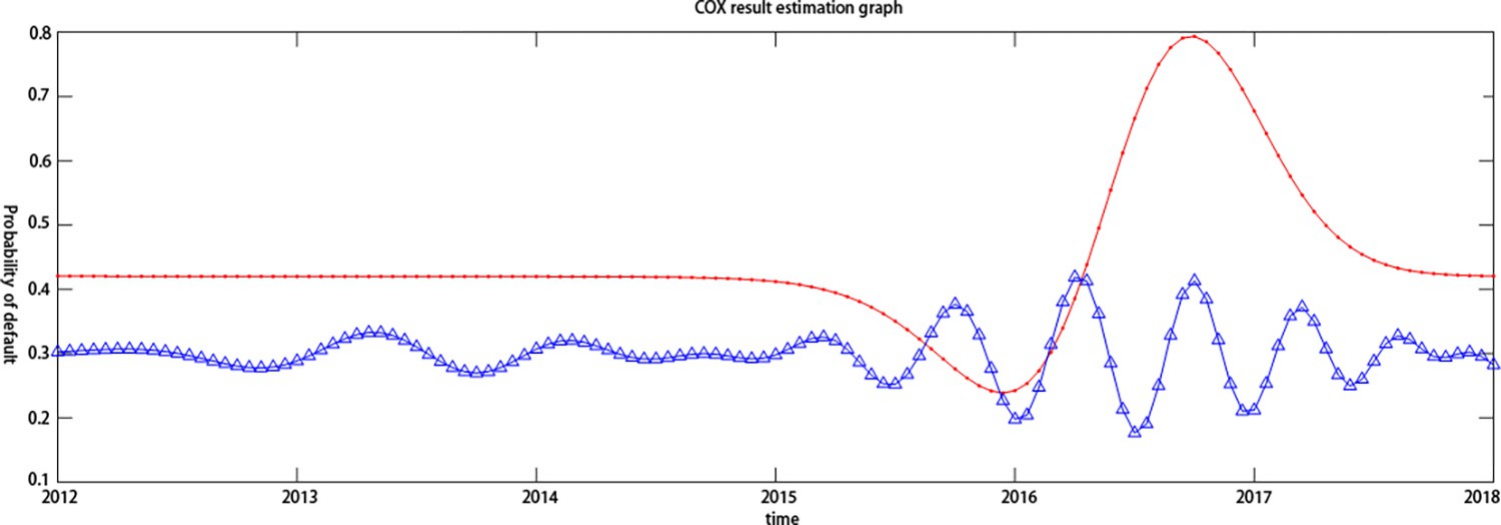
Synchronization of credit risk in the internet software industry. Note:Red Line: Tianxia Wisdom;Blue line: Haoyun Technology.

By introducing the cMCP-COX model, this paper compares the default probability synchronicity of the two listed companies, Eastcom Peace and Eastern Communications, from 2008 to 2018, and further judges the contagion of credit risk. At the same time, it compares Tianxia Wisdom and Haoyun Technology. The default probability of listed companies from 2012 to 2018 is synchronized to analyze the impact of the industry on the contagion effect. It can be seen from Fig 2. that the credit risk of the two companies in the electronic information technology industry increased and decreased simultaneously during the period of 2012–2014, which indicates that the credit risk levels of the two listed companies, Eastcom Peace and Eastern Communications, to a certain extent have a dependency relationship. Although the concealment of this dependency relationship was relatively high, the synchronization of this default probability makes it possible to transmit the credit risk to upstream and downstream companies in the supply chain. Oriental Credit’s credit risk continued to fluctuate between 2014 and 2018. This might be because Eastern Communications, downstream on the supply chain, is extremely susceptible to market uncertainties, so the credit risk also shows instability as a supply chain. The upstream core company Eastcom Peace stabilized its credit risk between 2014 and 2018, which further indicates that the core companies in the supply chain financial business process have lower credit risk due to their larger assets and better credit quality. As shown in Fig 3. the two listed companies in the Internet software industry also showed a trend of rising and falling at the same time in 2016–2017, indicating that the credit risk level still had a certain dependence in different industries, further confirming the credit risk contagion. Secondly, the level of credit risk fluctuations of Haoyun Technology in 2014–2018 is significantly lower than that of Eastern Communications, which indicates that the credit risk of different industries has different fluctuation trends. As part of the electronic information technology industry, Oriental Credit’s credit risk has greater volatility, which might be due to the industry as an outward-looking industrial cluster that is more susceptible to uncertain factors. Moreover, the Haoyun Technology of the Internet software industry is subject to The degree of influence of market uncertainties is relatively small, which indirectly proves that the industry is an important factor affecting credit risk and contagion effects. To further study the credit risk contagion of upstream and downstream companies in the supply chain, this article further evaluates the credit risk contagion by introducing the Copula model.

### 4.3 Determination of the edge distribution of the copula model

If the edge distribution functions F_1_ (x_1_), F_2_ (x_2_),…, F_n_ (x_n_) are uninterrupted, then the Copula function can be determined. Therefore, the first step in selecting the Copula function is the edge distribution, so that we can better fit the sample data to make the model more accurate. There are two methods to determine the edge distribution: the parametric method, whose steps include assuming the distribution of the variables, estimating the parameters based on sample values, and testing the model; and the non-parametric method, which involves using the sample empirical distribution or kernel density estimation to determine. In this paper, the functions jbtest, kstest, and lillietest were called in Matlab software. The Jarque-Bera test, Kolmogrov-Smirnov test, and Lilliefors test were performed on Eastcompeace and Dongfang Communication to evaluate the normality of the two listed companies.

**Fig 4:**
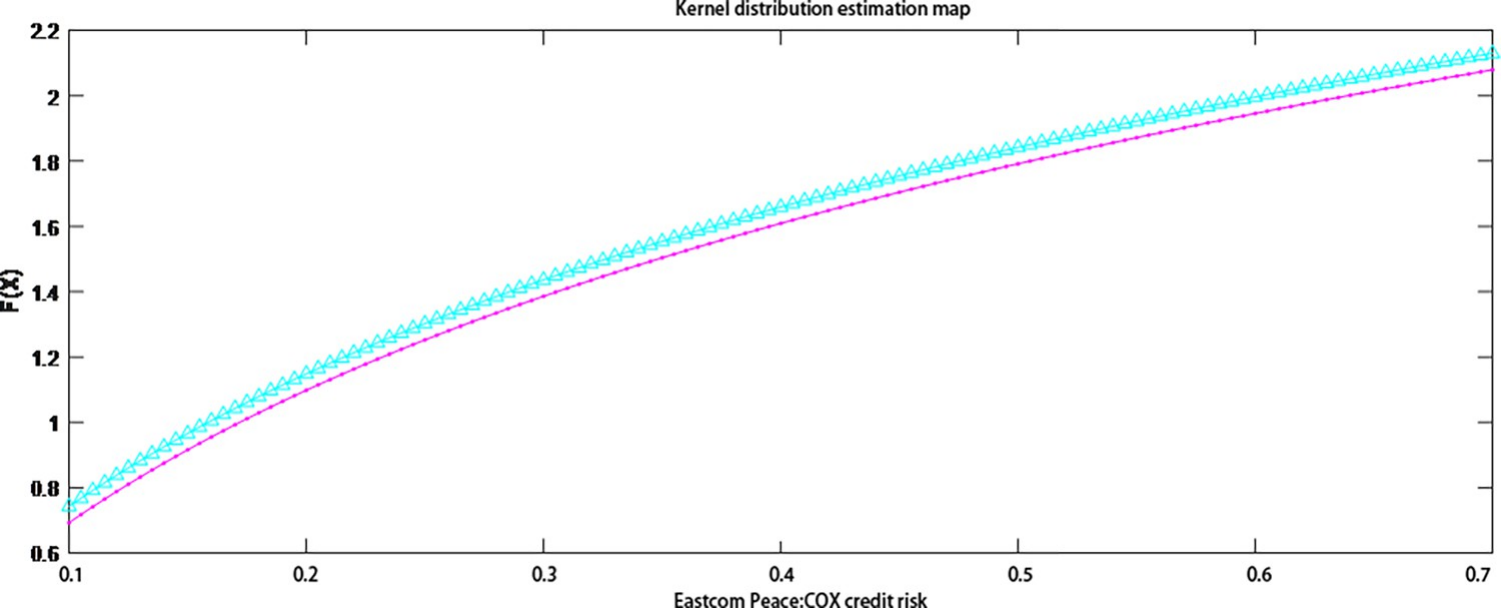
Estimation of the core distribution of Eastcompeace’s credit risk. Note: Red line: empirical distribution function; blue line: kernel distribution estimate.

**Fig 5:**
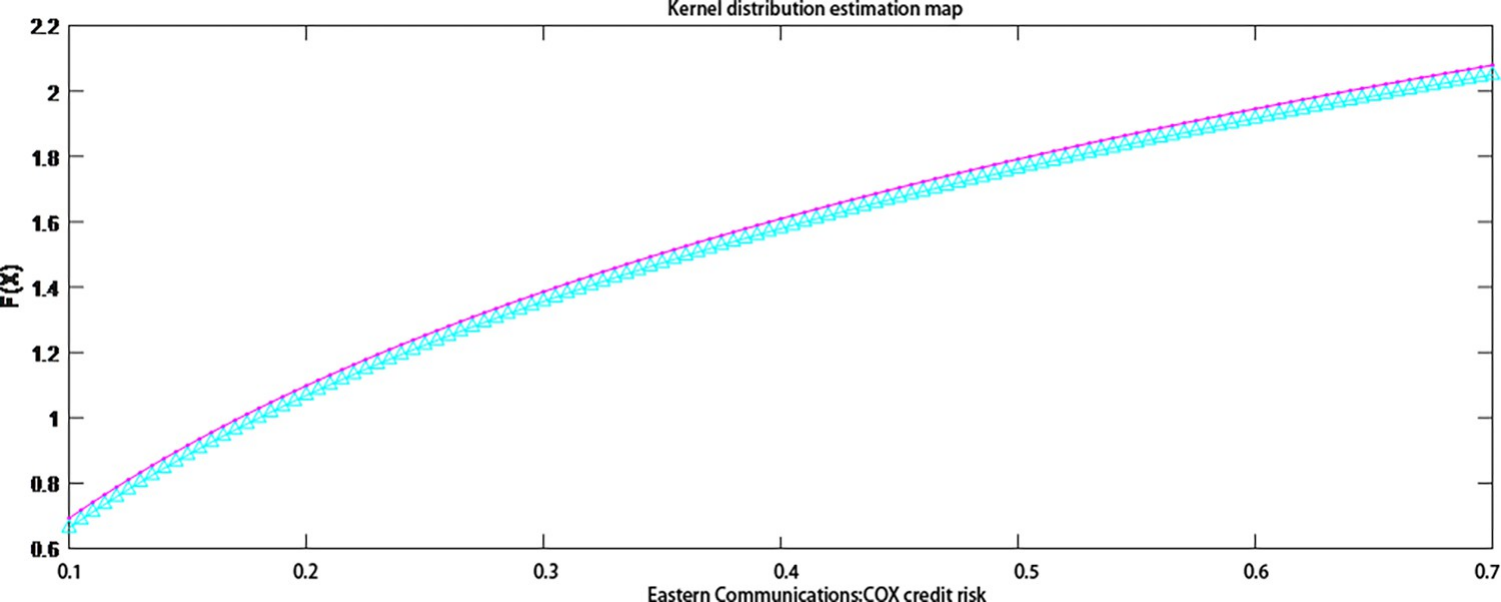
Estimation of the core distribution of Oriental Communication’s credit risk. Note: Red line: empirical distribution function; blue line: kernel distribution estimate.

It can be seen from Table 5. that the normality tests for Dongxin Heping and Dongfang Communication using different testing methods both have *h* values of 1 and *p* values less than 0.01, indicating that Dongxin Heping and Dongfang Communication are not characterized by a normal distribution. Therefore, this paper uses a kernel density estimation to make an accurate estimate of the credit risk distribution of the two listed companies. In this paper, the nuclear density estimation was performed in a Matlab environment and the nuclear distribution estimation charts for Eastcompeace and Dongfang Telecom were drawn (see Figs 4 and 5). The figure compares the nuclear and empirical distributions of the credit risk of the two listed companies. The change trend of the two is basically the same, which indicates that the goodness of fit of the core estimation distribution to the credit risk of the two listed companies is relatively high.

**Table 5:** Normality test results.

	Skewness	Kurtosis	JB test h value (P value)	KS test h value (P value)	Lilliefors test h value (P value)
Eastcom Peace	0.9392	3.2811	1 (1.0000e-04)	1 (3.1153e-21)	1 (1.0000e-05)
Eastern Communications	0.5624	3.1209	1 (1.0000e-04)	1 (3.2605e-04)	1 (1.0000e-06)

### 4.4 Determination of the copula model

We establish a Copula model to measure the correlation between variables. The main link is to choose the appropriate Copula function to characterize the relevant structure at the end of the data. Because the financial data of upstream and downstream companies in the supply chain have obvious “spikes, thick tails,” the correct Copula function is particularly important for data results. Because there are many types of Copula functions, different Copula functions can cause huge differences in the calculation results. Improper Copula functions might cause large errors in the empirical results or even incorrect conclusions. Therefore, choosing the correct Copula function becomes a key step in data analysis.

When describing the correlation structure between variables, different Copula functions have their own characteristics. The difference between the time-varying normal Copula function and the time-varying T-Copula function is that the tail of the time-varying T-Copula function has the strongest correlation and the time-varying function is positive. The stateful Copula function follows closely behind. The distributions of the time-varying Gumbel Copula function and the time-varying Clayton Copula function are not symmetrical; they are “J” and “L” type, respectively, and can describe the asymmetry of the correlation between the credit risks of the two listed companies. The difference is that the time-varying Gumbel Copula function is more sensitive to changes in the correlation of the credit risk of the two listed companies at the upper tail, while the time-varying Clayton Copula function is more sensitive to the change in the correlation at the lower tail. The time-varying layered Copula function has a great advantage in capturing the correlation between the upper and lower tails. Therefore, this paper chooses the aforementioned Copula function to describe the structure of Eastcompeace’s and Oriental Communications’ credit risk and selects the most suitable Copula function from those available. Parameter estimation results are shown in Table 6.

**Table 6:** Copula model parameter estimation results and square Euclidean distance.

Function type	Time-varying normal Copula	Time-varying T-Copula	Time-varying Gumbel Copula	Time-varying Clayton Copula	Time-varying stratification Copula
Parameter value	0.9067	3.4529	3.3321	4.8521	3.1451
d^2^	0.5882	0.7296	0.8845	0.6325	0.2926

Finally, Matlab software was used to calculate the Euclidean distances between the five time-varying Copula functions and the empirical Copula distribution functions (see Table 5. for the results) to evaluate the applicability of the model. The smaller the squared Euclidean distance is, the more suitable the model and the better the goodness of fit. Observing the value of the test statistic d^2^ in Table 6. we can see that there are differences in the goodness of fit of different Copula functions when describing the correlation structures and degrees of correlation between the two sequences. The time-varying normal Copula function is next. The time-varying Gumbel Copula function had the worst fit to the sample. Therefore, this paper considers that the correlation structure between Eastcompeace’s and Oriental.com’s credit risk should be fitted using a time-varying, layered Copula function.

### 4.5 Estimation of the dependent structure of credit risk tail in supply chain enterprises

Based on the covariant time-varying layered Copula model constructed above, we examine the credit risk contagion effect of upstream and downstream companies in the supply chain through the lower-tail dependence of credit risk. Table 7. gives the estimated results of the bottom-end dependence coefficients of the credit risk of the two companies, Eastcom Peace and Eastern Communications. From this, we can learn that the credit risk contagion effect of upstream and downstream companies in the supply chain has the following characteristics: the Copula model’s lower-tail dependence coefficient of credit risk is higher than the macro and company micro-financial covariate Copula model lower-tail dependence coefficient. As shown in Table 6. the dependence coefficient between Eastcom Peace and Eastern Communications after adding macro and company micro-financial covariates is 0.615, the standard deviation is 0.832, and the parameter acceptance probability is 0.432. The tail dependence coefficients of the macro and company micro-financial covariate models indicate that the credit risk transmission between enterprises is hidden. From the appearance characteristics, we might not be able to judge whether the credit risk of upstream and downstream companies in the supply chain has contagion effects. But when we consider the macro-level economic environment or micro-level characteristics of the enterprise, we can use a model to characterize the possibility of credit risk contagion between upstream and downstream companies in the supply chain to a certain extent.

**Table 7:** Estimate of tail dependence coefficient under credit risk.

	Model (1)	Model (2)	Model (3)
Mean	0.192	0.403	0.615
Median	0.103	0.292	0.832
Standard deviation	0.251	0.382	0.381
Acceptance probability	0.102	0.179	0.432

Note: Model (1) is a time-varying layered Copula model of macro covariates, model (2) is a time-varying layered Copula model of company microfinance covariates, and model (3) is a time-varying layering of macro-company microfinance covariates Copula model.

**Fig 6:**
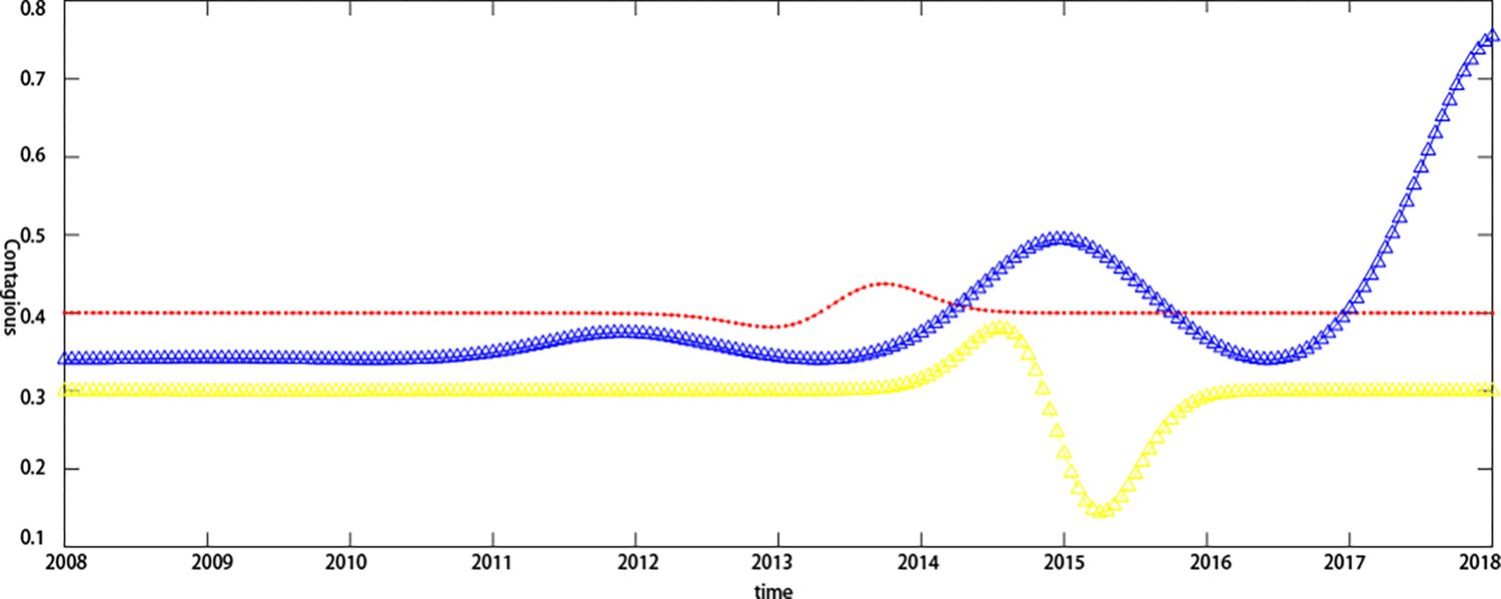
Dynamic characteristics of credit risk transmission in the electronic information technology industry. Note: Red line: Macro covariate model; Yellow line: Micro covariate model;Blue line: Macro and micro covariate model.

**Fig 7:**
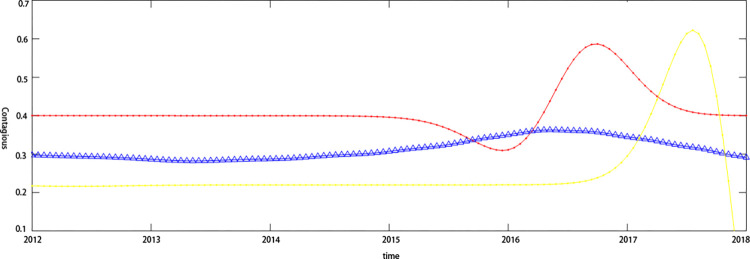
Dynamic characteristics of credit risk transmission in the Internet software industry. Note: Red line: Macro covariate model; Yellow line: Micro covariate model; Blue line: Macro and micro covariate model.

The credit risk contagion effects of upstream and downstream companies in the supply chain have dynamic characteristics that are subject to different trends influenced by the macroeconomic environment or the company’s micro-operational capabilities and financial condition. Fig 6. shows the dynamic characteristics of the credit risk contagion effect between the electronic information technology industry, and Fig 7. shows the dynamic effect of the credit risk contagion effect on the Internet software industry. From the estimation results of the macro-covariate model, it can be seen that the bottom-end dependence coefficient of the default distance of upstream and downstream companies in the Internet software industry’s supply chain is relatively high, indicating that under the influence of macroeconomic factors, credit risk contagion between Tianxia Wisdom and Haoyun Technology has occurred. The possibility of contagion effects is high. From the results of adding the company’s micro-financial covariate model and adding the macro-covariate and the company’s micro-financial covariate model to the bottom-end dependence coefficient of credit risk, it was found that the heterogeneity of the micro-capacity and financial status of upstream and downstream companies in the supply chain makes the model in this paper unable to capture the dynamics of the credit risk contagion effect. The tail dependence coefficient of the default distance is significantly lower than that of the macro-covariate model, which explains the possibility of the credit risk contagion effect occurring between enterprises.

For the analysis of the influencing factors of the credit risk contagion effect of upstream and downstream enterprises in the supply chain, this paper found that for the macroeconomic environment or the company’s micro-capacity and financial status, there are differences in the degree of influence and direction of these variables for credit risk contagion. To answer this question, we studied the impact of macro covariates and corporate micro finance covariates on the spread of credit risk. As shown in Table 8. gross domestic product (GDP) growth rate and broad currency growth rate have a positive effect on the contagion effect, while the consumer price index, short-term loan interest rate, and the exchange rate of renminbi (RMB) against the US dollar have a negative effect on contagion. Among the factors, the short-term loan interest rate has the greatest impact, and for the micro-financial situation, the financial ratio structure and enterprise qualification have a greater impact on the contagion effect of credit risk.

**Table 8:** Parameter estimation results of credit risk contagion model.

	Macro-covariate model	Micro-covariate model	Macro-Micro covariate models
Consumer Price Index	-0.101		1.287
GDP growth rate	0.044		-0.055
Broad money growth rate (M2 growth)	0.255		0.042
short term interest rate	-0.566		0.049
RMB/USD spot rate	-0.277		-0.485
Eastcom Peace:Solvency		0.027	-0.025
Eastcom Peace:Financial ratio structure		0.066	0.0005
Eastcom Peace:Cash flow level		-0.157	0.106
Eastcom Peace:Stock investment value		0.726	-0.319
Eastern Communications:Solvency		-0.143	-0.239
Eastern Communications:Financial ratio structure		0.356	0.092
Eastern Communications:Cash flow level		-0.617	0.99
Eastern Communications:Stock investment value		0.019	0.055
LPS	-142.215	-469.245	-491.208

## 5. Conclusion

This paper examines the credit risk measurement and contagion effects between upstream and downstream companies in the supply chain by constructing a Cox-Copula model and conducting an empirical analysis of the credit risk contagion of member companies in the industrial chain.

1. By introducing the COX model, this paper compares the default probability synchronicity of the upstream and downstream companies from two different industries to analyze the contagion of credit risk. The study reveals a dependency relationship between the upstream and downstream companies with synchronized credit risk trends indicating potential risk transmission in the supply chain, and also proves that the industry is an important factor affecting credit risk and contagion effects.
2. The credit risk contagion effect between the supply chain enterprises has dynamic characteristics which are subject to different trends influenced by the macroeconomic environment, the company’s micro-operational capabilities and financial conditions, based on the covariant time-varying layered Copula model. Furthermore, these factors show differences in the degree and direction of influence on the contagion effect, which has been analyzed between two different industries.
3. This paper shows that the selection of Cox-Copula model is appropriate to assess the credit risk and contagion effects of upstream and downstream companies in the supply chain, due to the heterogeneity of the relationship between the supply chain companies. The Cox model does not need to establish a model for each survival time and can analyze time as a continuous variable, which is more accurate in assessing the credit risk of supply chain companies. The Copula model can capture the correlation between variables because of its tail-dependent features, which is more suitable for studying the contagion problem of credit risk.
4. If supply chain companies are susceptible to changes in the international and domestic macroeconomic environment, if they are not guarded, the “domino” type of credit risk contagion effect caused by corporate associations might increase the probability of corporate credit default, causing potentially significant losses in the credit assets of financial institutions.

In the research process, this paper focused on the continuity between covariates and the asymmetrical correlation between variables, which greatly enhanced the stability of the prediction results. However, research on the characteristics of dynamic changes between covariates that are susceptible to accidental events might have certain limitations. In further research, factors such as accidental contagion of supply chain financial credit risk will be introduced and processing methods such as long memory effect will be used in the model to further enhance its theoretical and practical application value.

## 6. Contribution and application

This paper contributes to the understanding of credit risk measurement and contagion effects within supply chain networks through the construction and empirical analysis based on the Cox-Copula model. Specifically, it investigates the dependency relationship and potential risk transmission in the supply chain by comparing default probability synchronicity of upstream and downstream companies across different industries, revealing industry influence on credit risk contagion. Furthermore, it explores the dynamic characteristics of credit risk contagion, considering macroeconomic environments and company-specific operational and financial conditions and analyzes the varied impact of these factors on contagion effects between different industries.

In terms of applications based on this paper, the financial institutions can apply the Cox-Copula model to evaluate the creditworthiness of SMEs lacking collateral, enabling them to access capital for business development and thereby stimulating overall economic growth. Enterprises can regard the analysis as a reference to manage their business capabilities and financial status effectively by adjusting management strategies, production plans, and financing arrangements, thereby mitigating the probability of credit risk transmission among enterprises. Governments are also encouraged to implement measures such as monetary policy adjustments to stabilize the macroeconomic environment, fostering a conducive market atmosphere for business operations.
